# Prophylactic effects of nutrition, dietary strategies, exercise, lifestyle and environment on nonalcoholic fatty liver disease

**DOI:** 10.1080/07853890.2025.2464223

**Published:** 2025-02-12

**Authors:** Xiangyong Hao, Hao Song, Xin Su, Jian Li, Youbao Ye, Cailiu Wang, Xiao Xu, Guanglong Pang, Wenxiu Liu, Zihan Li, Tian Luo

**Affiliations:** a Department of General Surgery, Gansu Provincial Hospital, Lanzhou, China; b Department of clinical medicine, The First Clinical Medical College of Gansu University of Chinese Medicine (Gansu Provincial Hospital), Lanzhou, China; c The Institute for Clinical Research and Translational Medicine, Gansu Provincial Hospital, Lanzhou, China

**Keywords:** Nonalcoholic fatty liver disease, nutrition, dietary strategies, exercise, lifestyle, environment, prevention

## Abstract

**Background:**

Nonalcoholic fatty liver disease (NAFLD) is a chronic liver disease and its prevalence has risen sharply. However, whether nutrition, dietary strategies, exercise, lifestyle and environment have preventive value for NAFLD remains unclear.

**Methods:**

Through searching 4 databases (PubMed, Web of Science, Embase and the Cochrane Library) from inception to January 2025, we selected studies about nutrition, dietary strategies, exercise, lifestyle and environment in the prevention of NAFLD and conducted a narrative review on this topic.

**Results:**

Reasonable nutrient intake encompassing macronutrients and micronutrients have an independent protective relationship with NAFLD. Besides, proper dietary strategies including mediterranean diet, intermittent fasting diet, ketogenic diet, and dietary approaches to stop hypertension diet have their inhibitory effects on the developmental process of NAFLD. Moreover, right exercises including walking, jogging, bicycling, and swimming are recommended for the prevention of NAFLD because they could effectively reduce weight, which is an important risk factor for NAFLD, and improve liver function. In addition, embracing a healthy lifestyle including reducing sedentary behavior, not smoking, sleeping well and brushing teeth regularly is integral since it not only could reduce the risk of NAFLD but also significantly contribute to overall prevention and control. Finally, the environment, including the social and natural environments, plays a potential role in NAFLD prevention.

**Conclusion:**

Nutrition, dietary strategies, exercise, lifestyle and environment play an important role in the prevention of NAFLD. Moreover, this review offers comprehensive prevention recommendations for people at high risk of NAFLD.

## Introduction

Nonalcoholic fatty liver disease (NAFLD) is now established as the most prevalent chronic liver disease in the world [1,2]. The prevalence of NAFLD has significantly increased year by year, rising from 25.5% (20.1–31.0%) in or before 2005 to 37.8% (32.4–43.3%) in 2016 or later [3]. Besides, NAFLD impacts the health of 25% of the general population, with an even higher prevalence of 70% among individuals who are obese or have type 2 diabetes [4].

Moreover, NAFLD encompasses a range of disease processes, including non-alcoholic simple hepatic steatosis, which could progress to non-alcoholic steatohepatitis (NASH) and is classified by the severity of fibrosis, ranging from mild (F0–F1) to significant (≥ F2), advanced (≥ F3, bridging) or cirrhosis (F4), eventually leading to liver failure or cancer [5,6]. In addition, many metabolic risk factors such as overweight, obesity, diabetes mellitus, sarcopenia and dyslipidemia are associated with NAFLD in the majority of patients [7]. Therefore, comprehensive management strategies and interventions are needed to address the increasing burden of NAFLD.

Nutrition, dietary strategies, exercise, and lifestyle, considered as controllable risk factors for NAFLD, are deemed safer and more effective in preventing the onset of NAFLD compared to pharmacological treatments and bariatric surgery [8]. Firstly, dietary nutrients (including macronutrients and micronutrients) and healthy dietary strategies (including mediterranean diet, intermittent fasting diet, ketogenic diet, and dietary approaches to stop hypertension diet) have been shown to have an independent protective relationship with NAFLD [9]. Secondly, the most effective way to prevent NAFLD is to lose weight, so it is not surprising that more exercise targeting obesity are also used to prevent NAFLD [10]. Moreover, lifestyle factors encompassing moderate alcohol consumption, abstinence from smoking, reduced sedentary time and adequate sleep are regarded pivotal in the prevention of NAFLD due to its inverse correlation with the prevalence of the condition [11]. In addition, the social and natural environment is reported to be associated with various causes of liver diseases [12].

However, most studies on NAFLD prevention are single-center studies, while providing valuable insights, and there are limitations including small sample sizes, limited patient diversity, and potential selection bias which reduce the generalizability of the findings. Therefore, it is needed to carry out this review to make a more comprehensive exploration and evaluation on prophylactic effects of nutrition, dietary strategies, exercise, lifestyle as well as social and natural environment on NAFLD based on previous studies.

This review provides a new perspective on NAFLD prevention by integrating the results of different studies and emphasizing the intrinsic mechanisms of nutrition, dietary patterns, exercise, lifestyle, and the environment in the prevention of NAFLD. Furthermore, this review could not only offer feasible recommendations for preventing NAFLD in relevant populations, but also provide valuable guidance for future research and clinical practice.

## Methods

Through searching 4 databases (PubMed, Web of Science, Embase and the Cochrane Library) from inception to January 2025, we selected studies about nutrition, dietary strategies, exercise, lifestyle and environment in the prevention of NAFLD and conducted a narrative review on this topic. In addition, the following MeSH terms and/or free text terms were used as search terms: ‘Nonalcoholic fatty liver disease’, ‘nutrition’, ‘dietary’, ‘exercise’, ‘lifestyle’, ‘environment’.

## Different strategies for preventing NAFLD

### Prophylactic effects of nutrition on NAFLD

#### Macronutrients

##### Carbohydrates

It is well known that carbohydrates, although an important source of energy, could have harmful effects on liver health [13]. Currently, fructose, glucose and sucrose are the most consumed sugars, while refined carbohydrates, which are usually abundant in modern diets, contribute to intrahepatic triglyceride (IHTG) deposition, and excessive intake promotes de novo lipogenesis (DNL), both of which play a role in the pathogenesis of NAFLD [14]. Besides, in a large cohort study (*n* = 4365), it was found that individuals with NAFLD had higher intake of carbohydrates and free sugars compared to participants without NAFLD [15]. However, the reduction in carbohydrate consumption is most directly related to refined carbohydrates, particularly those high in glycemic index, as opposed to all carbohydrates in general [16]. Evidence suggested that a low-carbohydrate diet could be effective in reducing body weight and body fat in patients with NAFLD, while also improving metabolic indicators such as liver enzymes, blood glucose, blood lipid, and uric acid levels [17]. Moreover, there is also evidence indicates that an energy-restricted diet with low carbohydrate content (total energy intake of 90 g carbohydrates and at least 30% fat) may be effective in preventing or treating NAFLD, which is beneficial to improve metabolic parameters in patients with NAFLD [18]. In addition, it has been shown that female NAFLD patients exhibited more significant improvements in these indicators than male patients [19]. Besides, researchers have suggested that the avoidance of high carbohydrate intake should be strongly recommended to diminish fat accumulation in the liver [8].

The above evidences suggested that low carbohydrate diet contributes to the prevention of NAFLD. However, a low carbohydrate diet seems to be more effective in reducing liver fat, but it is only in the short term [20]. Therefore, further studies are needed to explore the mechanisms of carbohydrate on NAFLD and to make a sustainable dietary recommendation.

###### Fructose

As a monosaccharide, fructose is usually consumed in a diet rich in both glucose and lipids [21]. Moreover, the global increase in fructose consumption is largely attributed to the widespread availability and affordability of high-fructose corn syrup, which is a key ingredient in many sweetened beverages and processed foods [22].

There is an evidence that under isocaloric conditions, the type of carbohydrate consumed is the most relevant factor for the prevalence of NAFLD, such as fructose consumption has been linked to increased IHTG, particularly when consumed in liquid form [23]. According to some epidemiological studies, a sharp increase in fructose consumption is strongly associated with an increase in NAFLD incidence [21,24,25]. Evidence indicates that excessive fructose consumption is known to promote NAFLD, since fructose is both a substrate and an inducer of hepatic DNL [21]. Furthermore, in hepatocytes, fructose bypasses the rate-limiting step of glycolysis catalyzed by phosphofructokinase, potentially providing more substrate than glucose for the DNL pathway and IHTG accumulation [26]. In addition, it was showed that excessive fructose consumption with several key mediators released by the liver leads to altered communication between the liver and the intestinal tract, muscle and adipose tissue, and leads to exacerbation of NAFLD [27]. Therefore, effective management of fructose intake, along with a conscious choice to limit consumption of fructose-containing drinks and foods, could contribute to the prevention of NAFLD.

###### Fiber

Dietary fibers, which are helpful in preventing NAFLD, could be categorized into two types: soluble fibers and insoluble fibers [28]. Soluble fibers, such as beta-glucans and psyllium, contribute to NAFLD prevention by reducing cholesterol absorption, enhancing glycemic control, and modulating lipid metabolism [29]. Besides, insoluble fibers, including wheat bran, primarily improve gastrointestinal motility, which indirectly mitigates systemic inflammation and promotes metabolic homeostasis, factors critical to liver health [30]. A review indicates that a high-fiber diet (50 g/day of both soluble and insoluble fibers) could lower the average daily blood glucose concentration (*P* < 0.05), decrease hypoglycemic events (*P* < 0.01), and reduce glycated hemoglobin levels (*P* < 0.05) in patients adhering to the diet regimen [31]. Similarly, considering the high prevalence of NAFLD in patients with dyslipidemia, type 2 diabetes, and metabolic syndrome, incorporating fibers as a therapeutic strategy for glycemic, lipid, and weight control in individuals with steatosis seems reasonable [32]. Moreover, it was recommended that the daily intake of fiber is between 20 g/day and 40 g/day, with 5–15 g/day coming from soluble fibers [33,34].

In summary, with the increasing prevalence of highly processed and refined foods in modern diets, global dietary fiber intake is on a downward trend. Therefore, the promotion of high-fiber foods is a key step in reducing the incidence of NAFLD worldwide.

###### Nuts

Nuts are a rich source of nutrients and contain many health-promoting compounds, including macronutrients (mainly unsaturated fatty acids and high-quality proteins), micronutrients (minerals, water-soluble vitamins such as folic acid and fat-soluble bioactive) and cellulose [35,36]. The unsaturated fatty acids in nuts, primarily monounsaturated and polyunsaturated fats, contribute to improved lipid profiles, reduced inflammation, and enhanced insulin sensitivity, which are critical factors in the prevention and management of NAFLD [37]. Additionally, the high-quality proteins in nuts play a role in satiety and metabolic regulation, potentially influencing body weight and energy balance, both of which are relevant to NAFLD prevention [38].

There are some epidemiological studies have reported that a direct link between continuous nut consumption and reduced levels of inflammation, insulin resistance, oxidative stress, and metabolic syndrome, all of which are involved in the pathogenesis of NAFLD [39–41]. Furthermore, a cross-sectional study (*n* = 1848) of nut intake in older adults suggested that moderate nut intake (15.1–30.0 g/day) was associated with fatty liver index, but the association was not statistically significant when some possible confounding factors were considered (*P* = 0.329) [42]. However, it was reported that the association between daily nut intake and NAFLD risk showed a significant positive correlation in deciles 9 and 10 compared to the lowest decile (odds ratio [OR]: 3.22, 95% confidence interval [CI]: 1.04–7.49, *P* = 0.039 and OR: 3.03, 95% CI: 1.03–8.90, *P* = 0.046, respectively) in a case-control study [43].

Despite these findings, the relationship between nut consumption and NAFLD remains inconclusive, with potential differences arising from nut type, quantity, and accompanying dietary patterns. Further researches are warranted to clarify the impact of nuts’ macronutrient composition, including fats and proteins, on NAFLD incidence and progression.

##### Protein

Protein constitutes a significant constituent of dietary nutrients, accounting for 10% to 35% of total daily caloric intake, so it is crucial to ensure that patients fulfill their protein requirements to preserve lean body mass [44,45]. It has been suggested that increasing dietary protein content attenuates the effect of hypercaloric feeding with fructose or fat on IHTG levels [46]. Besides, it was reported that high-protein (HP) intake reduced the weight loss induced decline in lean tissue mass by 45% [47]. Moreover, there is evidence that HP intake combined with low carbohydrate diet improves carbohydrate metabolism and liver steatosis by inhibiting adipose neogenesis [48]. Additionally, the European Society for Clinical Nutrition and Metabolism guidelines recommend a protein intake of 1.2–1.5 g/kgBW/d [49]. Furthermore, the subsequent step in optimizing nutrition care involves discussing the source of protein once an individual meets the aforementioned nutritional recommendations [44]. High-quality protein could be obtained from plant-based and dairy sources (milk, grains, vegetables, and protein powders), which high-risk individuals are encouraged to consume [44]. Therefore, the intervention of protein intake as a measure of prevention of NAFLD has been widely recognized.

##### Fat

A high-fat diet, usually defined as consuming more than 35% of the total daily calorie intake comes from fat, is widely recognized as leading to obesity and hepatic steatosis [50,51]. A mouse model has demonstrated that dietary fat, rather than carbohydrate or protein, is the primary driver of increased energy consumption and adiposity [52]. Moreover, a prospective study found that a 3 weeks high-fat diet leads to intrahepatic lipid accumulation and a decreased metabolic flexibility (change in respiratory quotient was +0.02 ± 0.02 *vs.* −0.05 ± 0.1 in low-fat *vs.* high-fat group, *P* = 0.009) [53]. Additionally, it is recommended that the daily dietary fat intake of healthy adults should be 20–35% of the total calorie intake [34]. Therefore, reducing fat consumption in the daily diet could reduce obesity rates and subsequently prevent NAFLD.

###### Saturated and unsaturated fatty acids

Saturated fatty acids (SFA) are known to have a negative impact on lipid and glucose balance, aggravating the development of metabolic syndrome and increasing the risk of NAFLD [54,55]. Besides, it was found that excessive consumption of dietary SFA and cholesterol synergistically induces the metabolic and hepatic features of NASH in a mouse model [56]. However, although a diet containing 8–10% SFA could be beneficial, a drastic reduction in SFA (< 6%) may have a detrimental effect on plasma lipid levels [8].

In contrast, unsaturated fatty acids, particularly polyunsaturated fatty acids (PUFAs) such as omega-3 fatty acids, are associated with a protective effect against NAFLD [57,58]. Besides, it was found that omega-3 fatty acids are associated with a significant reduction in gamma-glutamyltransferase activity, decreased liver fat in weight losers, and beneficial changes in plasma lipid profiles in a double-blind placebo-controlled trial (*n* = 60) [58]. Moreover, PUFAs could reduce hepatic fat accumulation by enhancing fatty acid oxidation, improving insulin sensitivity, and suppressing de novo lipogenesis [57].

In conclusion, dietary recommendations for NAFLD prevention should emphasize replacing SFA with unsaturated fats while maintaining overall dietary balance. Future researches are essential to determine the optimal levels and ratios of saturated and unsaturated fats to minimize NAFLD risk and promote metabolic health.

###### Trans fatty acids

Trans fatty acids (TFA) are mainly derived from hydrogenated oils such as margarine and other processed foods, and their global intake increases with the widespread supply of processed foods [59]. However, dietary TFA do not originate only from processed foods, but also occur naturally in small amounts in ruminant products such as dairy and meat [60]. A clinical trial has demonstrated the adverse effects of trans fats in food on human health and nutrition, high intake of TFA could increase the risk of NAFLD [61]. Although its underlying mechanism has not yet been fully established, intake of a large amount of TFA from hydrogenated oil will increase the level of inflammatory markers and lead to endothelial dysfunction and harmful changes in lipid profiles (increased LDL/HDL and TC/HDL ratios) [62]. To mitigate these risks, it is recommended that TFA intake should constitute less than 1% of daily calorie intake [63]. Collectively, reducing dietary intake of TFA through public health interventions such as banning industrial TFA and reformulating processed foods could be an important strategy for preventing NAFLD.

###### Lard and soybean oil

The unsaturated fatty acids in soybean oil are mainly omega-6 fatty acids [64], which are beneficial to the NAFLD, but prolonged intake of large quantities may enhance cholesterol-induced mitochondrial damage, which leads to hepatocyte death, recruitment of inflammatory cells and fibrosis, and led to a transition from steatosis to NASH, doubling the NASH activity score [65]. Moreover, lard is regarded as SFA and its consumption has decreased significantly in recent years due to its increase in low-density lipoprotein cholesterol [66].

However, recent studies have shown that the combination of lard and soybean oil may have the potential to alleviate NAFLD compared with the adverse effects of butter and margarine on NAFLD [66,67]. For instance, an experimental study has demonstrated that mixed oil of lard and soybean oil significantly inhibited serum triglyceride, liver triglyceride, serum free fatty acids (FFAs) and liver FFAs in mice [67]. Similarly, the results of liquid chromatography-mass spectrometry showed that the mixed oil promoted the binding of bile acids to taurine and glycine, thereby activating G-protein-coupled bile acid receptor 1 for improved lipid metabolism [67].

The above researches indicated that the combination of lard and soybean oil could inhibit hepatic fat accumulation and improve lipid metabolism, offering new insights for the prevention of NAFLD. Therefore, the combination of lard and soybean oil is deemed to possess therapeutic properties, making it a promising dietary intervention for individuals with NAFLD by increasing the intake of this mixture.

#### Micronutrients

##### Prebiotics and probiotics

Prebiotics are indigestible carbohydrates that stimulate the growth and activity of probiotics (beneficial bacteria), mainly lactic acid bacteria and bifidobacterial [34]. They also have been utilized to prevent NAFLD due to their capacity to directly and indirectly modify the local microbiota, enhance the formation of the epithelial barrier, and reduce intestinal inflammation and oxidative stress [68]. Moreover, several studies have demonstrated that different prebiotics could reduce the prevalence of NAFLD [69–71]. For instance, there’s a potential role for metronidazole, combined with inulin, in modulating gut microbial function (e.g., metabolite production), which is suggested to alleviate steatohepatitis [71]. Furthermore, alanine aminotransferase (ALT) levels decreased significantly after treatment with metronidazole-inulin compared to after receiving placebo-placebo (mean ALT change of −19.6 *vs.* −0.2 U/L, respectively; *P* = 0.026) [71].

Besides, fructo-oligosaccharides and inulin derived from natural sources such as onions, garlic, wheat, rye, chicory roots, unlike fructose in processed foods and beverages, have shown promise for improving NAFLD [72,73]. It was suggested that inulin could restore gut microbiota, which leads to the amelioration of NAFLD and the mechanism behind this restoration may be related to increased butyrate production, reduced endotoxemia, and decreased lipogenesis [73]. Furthermore, the consumption of white kidney beans (WKB) not only normalized dysbiosis in the intestinal flora of mice but also demonstrated effectiveness in reducing obesity, lipids, and blood glucose levels in an intervention study [74]. Additionally, WKB improved markers of insulin resistance and liver function, ultimately contributing to the alleviation of NAFLD [74].

In conclusion, supplementation with probiotics or restoration of intestinal flora and supplementation with commensal bacterial metabolites may have therapeutic benefits for NAFLD.

##### Choline

Choline is an essential nutrient that plays a crucial role in proper liver, lipid metabolism, cellular membrane composition and repair [75]. The primary dietary sources of choline include eggs, fish, grains, meat, milk and its derivatives, along with vegetables such as soybeans (whole beans) and potatoes [76]. Moreover, it has been reported that the gut microbiota metabolizes choline to produce trimethylamine, which is then converted into trimethylamine-N-oxide in the liver upon host absorption, the high levels of both compounds are associated with NAFLD [77]. Similarly, an animal study using a methionine/choline-deficient diet to develop a NASH model found that choline deficiency could cause NAFLD [78]. Therefore, choline has the advantage of being readily available in the diet and increasing the number of choline-rich foods, which provides a new idea for the prevention of NAFLD.

##### Palmitic acid

Palmitic acid is an SFA, which is abundant in palm oil, and its fatty acid content is higher than that of other vegetable oils, and regular consumption of palm oil has been associated with increased accumulation of body fat [79,80]. Moreover, it was showed that in cells exposed to palmitic acid, obvious fat accumulation morphology could be recorded by oil red O staining, and intracellular triglyceride levels increase [81]. Importantly, it was reported that palmitic acid could induce a significant increase in the level of interleukin-8, a bioactive neutrophil chemoattractant, in steatosis hepatocytes, which may lead to liver inflammation and subsequent liver injury [81,82]. Therefore, it is recommended to limit the intake of SFA in the diet because of the potential effects of palmitic acid on steatosis and hepatitis. Besides, more studies are required to investigate specific mechanisms of palmitic acid-induced NAFLD and recommended appropriate palmitic acid intake in the future, which will provide a deeper understanding for the development of methods for the prevention of NAFLD.

##### Vitamin B_12_

Vitamin B_12_ exists in two main forms in humans: 5′-deoxyadenosylcobalamine and methyl cobalamin, and its deficiency has been linked to the progression of NAFLD [83,84]. Besides, it was reported that vitamin B_12_ supplementation could decrease serum levels of homocysteine in patients with NAFLD in a randomized controlled trial (*P* = 0.038) [85]. Similarly, elevated homocysteine levels in the liver lead to its binding with a protein called syntaxin17 (STX17), inhibiting the protein’s function in fatty acid metabolism and fat digestion, which triggers the progression of fatty liver to NASH [86,87]. Additionally, vitamin B_12_ and folic acid supplementation could restore STX17 expression and its autophagic function and reverse hepatic inflammation and fibrosis in NASH, which may be used as a new therapy to prevent NASH [88].

Paradoxically, there is evidence that increasing serum concentrations of vitamin B_12_, even within physiological levels, may play a role in the risk of NAFLD [89]. Moreover, based on several studies, there is no significant difference in vitamin B_12_ levels between NAFLD patients and control subjects [90–92]. However, these findings may be limited by the small sample size used in the study [93].

In conclusion, while vitamin B_12_ may have a beneficial role in NAFLD management, further research with larger sample sizes, different doses, and types of vitamins B_12_ is required to establish this role definitively based on the preceding evidence.

##### Vitamin E

Vitamin E is a lipophilic compound that exists naturally in the form of tocopherol and tocotrienol [94]. Among these compounds, tocopherol stands out as the most abundant and potent antioxidant, possessing significant free radical-scavenging properties so that it was considered valuable in the prevention of NAFLD [95]. Therefore, the American Association for the Study of Liver Diseases and the National Institute for Health and Optimization recommend the use of vitamin E (800 IU/day) for the treatment of NAFLD, because vitamin E could be used as a natural antioxidant to improve steatosis, inflammatory responses, and reduce transaminase levels [96]. Furthermore, vitamin E is often used in combination with silymarin in the prevention of patients with NAFLD [97]. In addition, there is evidence that supplementation with 100–1200 IU/day of vitamin E for at least 24 weeks has been shown to improve liver biochemistries and histology [98]. Similarly, it was demonstrated that a dose of 800 IU/day of α-tocopherol vitamin E for 96 weeks was linked to reduced serum aminotransferases and histological improvement in steatosis, inflammation, and ballooning, as well as the resolution of steatohepatitis in adults with NASH [99].

To sum up, vitamin E continues to exhibit significant potential in enhancing both biochemical and histological markers of NAFLD, particularly when used as an adjunct therapy alongside other interventions such as lifestyle adjustments. Consequently, vitamin E should be more widely employed as an adjuvant medication for the treatment of NAFLD in clinical settings.

##### Iron

From a clinical perspective, excess iron has been found to worsen the natural progression of NAFLD due to its ability to catalyze the formation of toxic hydroxyl radicals, resulting in cellular damage [100]. Likewise, the accumulation of iron in NAFLD is primarily caused by the inhibition of iron mobilization in hepatocytes and Kupffer cells [101]. Conversely, iron deficiency may also influence NAFLD by relying on HIF2α-ATF4 signal transduction to enhance hepatocyte adipogenesis and insulin resistance [102]. Therefore, understanding the balance between iron deficiency and overload is crucial, as both conditions could exacerbate liver dysfunction in distinct ways.

Additionally, a prospective study revealed a significant interactive dialogue between intestinal flora, iron status, and liver fat accumulation, particularly within the microbiome-iron-liver fat axis [103]. Notably, it also suggested that dysbiosis may exacerbate iron overload by increasing intestinal luminal iron uptake or by affecting bacterial metabolites that regulate hepatic inflammation and fat deposition [103]. Similarly, it was reported that iron overload is harmful to NAFLD and the removal of excess iron may have some clinical benefits for specific patients with confirmed iron overload [101,104]. Therefore, monitoring the level of iron in the body has potential clinical application value and could be used as a promising way to prevent NAFLD.

##### Copper

Copper, as a trace element, has received attention in the prevention of NAFLD because of its dual role in deficiency and excess [105,106]. In fact, copper deficiency has been shown to impair the antioxidant defense system, which may exacerbate liver injury and contribute to the progression of NAFLD [105]. Specifically, it has been demonstrated that copper deficiency could hinder the activity of key enzymes involved in cellular defense, thereby promoting oxidative stress in hepatocytes, which is a key trigger for NAFLD pathogenesis [105]. Conversely, evidence from the U.S. population has shown that elevated serum copper levels may be associated with an increased risk of both the onset and progression of NAFLD and related metabolic disorders, including insulin resistance and dyslipidemia [106]. This observation underscores the paradoxical role of copper in liver disease, as both deficiencies and excesses of copper could lead to metabolic dysfunction [106]. Notably, altered copper bioavailability, which has been shown to predict early atherosclerosis, serves as a major cardiovascular risk factor in obese patients with hepatic steatosis detected by ultrasound [107].

In summary, copper imbalance may play a significant role not only in liver disease progression but also in the cardiovascular comorbidities commonly associated with NAFLD. Moreover, these findings emphasize the need for further research to clarify the exact mechanisms by which copper dysregulation contributes to the pathogenesis of NAFLD and its associated complications.

### Prophylactic effects of water and beverages on NAFLD

#### Water

Water contaminants such as microcystin, disinfection by-products, and heavy metals in surface, ground, and irrigation waters globally have gained widespread attention due to they have the potential to contribute to NAFLD [108,109]. Similarly, it was showed that perfluorooctanoic acid in contaminated drinking water has been associated with a reduction in the levels of inflammatory cytokines, which could promote the development of fatty liver [110]. In addition, there is evidence that there are two primary molecular mechanisms of NAFLD induced by water pollutants: (1) Pollutants in water resources induce lipid metabolism disorders in hepatocytes by altering lipid synthesis-related enzymes or inhibiting mitochondrial fatty acid β-oxidation-related enzymes through the peroxisome proliferator-activated receptor (PPAR)-α signaling pathway [111]. (2) Pollutants promote oxidative stress or endoplasmic reticulum stress, which leads to liver inflammation [111].

While above findings indicate the harmful potential of water pollutants, emerging researches highlight that the toxicity of contaminants is often dose-dependent [112]. Moreover, the World Health Organization (WHO) has set a provisional guideline value of 1 µg/L for microcystin in drinking water, suggesting that concentrations below this level are considered low risk for human health [112]. Therefore, efforts should aim to minimize contaminant levels in drinking water as much as possible, even below established guidelines, to ensure public health protection.

In conclusion, pollutants in water resources have a positive effect on the progress of NAFLD. In the future, it is government departments and public health workers that are supposed to strengthen the treatment of pollutants in water. Besides, some low-cost adsorbents and materials for removing heavy metals in water should also be vigorously developed to protect water resources and prevent NAFLD.

#### Green tea

Green tea is a functional food rich in polyphenolic catechins, polyphenols catechins and epigallocatechin gallate (EGCG) [113]. Besides, EGCG is a polyphenol formed by epigallocatechin and gallic acid ester, which is the most abundant antioxidant catechin in green tea [114]. Moreover, animal and cellular studies have shown that green tea and EGCG could offer protection against the initiation and progression of NAFLD by mitigating oxidative stress and its associated metabolic dysfunction, inflammation, fibrosis, and tumorigenesis [115,116]. In addition, some epidemiological studies suggested that the concurrent ingestion of vitamin C and sucrose with green tea may enhance catechin bioavailability [117,118]. However, the amount of sucrose should be carefully regulated to prevent excess carbohydrate intake, which could counteract the benefits of green tea. A safe daily sucrose intake of less than 10% of total energy intake is recommended, aligning with dietary guidelines, while frequent consumption of green tea (5–10 cups/day) remains essential to fully realize its benefits [117,118].

Nevertheless, there remains a scarcity of randomized controlled trials investigating the potential benefits of green tea components for NAFLD. Consequently, further researches are imperative to furnish direct evidence regarding the efficacy of green tea in thwarting the development and progression of NAFLD, as well as to establish specific recommended intakes of green tea for maintaining health.

#### Coffee

Coffee is believed to contain three primary compounds that play physiological roles: caffeine, diterpenoids (cafestol and carvacrol), and chlorogenic acid [119]. Furthermore, it was reported that caffeine attenuates the progression of liver fibrosis by preventing adhesion and activation of hepatic stellate cells, stimulating oxidation through the autophagy-lysosome pathway, and preventing the expression of connective tissue growth factor by modifying signaling pathways [119]. Moreover, diterpenes, such as caffeinol and carvacrol, protect the liver against the expression of inflammatory markers through antioxidant effects by reducing inflammatory effects [120]. Similarly, the antioxidants chlorogenic acid and uridine diphosphate glucuronosyltransferase have been shown to prevent lipid accumulation in hepatocytes, reduce inflammatory responses, and promote insulin sensitivity [121].

Subsequently, a systematic review had reported that coffee intake level > 3 cups/day was observed lower risk of NAFLD than < 2 cups/day, which suggest a potential dose-dependent association between coffee intake and NAFLD risk [122]. However, it was demonstrated that the risk reduction for ≥ 5 cups/day are generally smaller than for 3 or 4 cups/day, although still protective compared with no coffee, suggesting that there is likely a level beyond which increasing coffee consumption confers no further benefit [123].

In summary, controlling coffee intake within the recommended range of 3-5 cups/day may be a potential measure to prevent NAFLD. This approach includes reducing the intake of excessive drinkers, while encouraging people who do not drink coffee or drink less coffee to drink coffee in moderation to achieve a protective effect on NAFLD.

#### Yogurt

Conventional yogurt was fermented by cultured *Lactobacillus delbrueckii* subsp [124]. The beneficial effects of yogurt are attributed to its content of probiotics, particularly strains of Lactobacillus and Bifidobacterium, which modulate the gut microbiota [125]. Compared to milk, yogurt could influence the species of gut microbes and significantly reduces homeostasis model assessment of insulin resistance, which is beneficial to alleviate NAFLD [126]. There is evidence that yogurt is superior to milk in improving insulin resistance and liver fat in Chinese obese women with NAFLD and metabolic syndrome, possibly by improving lipid metabolism, reducing inflammation, oxidative stress, lipase and changing gut microbiota composition [127]. Furthermore, several prospective studies have shown that yogurt intake (> 80 g/day) is inversely associated with the risk of obesity, metabolic syndrome, and diabetes [128–130].

In conclusion, yogurt exerts significant effects on the gut microbiota by enhancing the abundance of beneficial bacterial genera, such as Lactobacillus and Bifidobacterium, while concurrently suppressing pathogenic species. These microbiota-modulating properties contribute to the mitigation of NAFLD pathogenesis through both direct and indirect mechanisms. Directly, yogurt consumption promotes improved hepatic lipid metabolism and a reduction in hepatic fat accumulation. Indirectly, it attenuates systemic inflammation, oxidative stress, and insulin resistance, thereby addressing key metabolic derangements that predispose to the development and progression of NAFLD.

#### Alcohol consumption

The relationship between alcohol consumption and liver health has been extensively studied, with heavy alcohol intake unequivocally linked to the development and progression of NAFLD. Chronic heavy alcohol consumption, typically defined as consuming > 14 drinks per week for women or > 21 drinks per week for men, is a well-established risk factor for liver fibrosis, cirrhosis, and hepatocellular carcinoma [131]. Moreover, a cross-sectional study involving 2,629 participants reported that the higher the number of drinks per week (≥ 8 drinks per week for women and ≥ 15 for men) and the increased odds of fibrosis in patients who drank less than the heavy drinking threshold [132]. Additionally, it was suggested that the net effect of non-heavy alcohol consumption (less than 20 g/day for women and less than 30 g/day for men) on the liver is also detrimental [133,134]. Besides, a cohort study (*n* = 8345) found that even low alcohol intake (0–9 g of alcohol per day) in fatty liver disease was associated with an increased risk of advanced liver disease and cancer [135].

In contrast, the impact of alcohol consumption on liver health has been controversial [136]. It was showed that non-heavy alcohol consumption, could even have some beneficial metabolic effects and is associated with a reduced risk of NAFLD [137,138]. Likewise, it was reported that daily small alcohol consumption (1–2 drinks per day or up to 10 g of alcohol per day) has a protective effect on the development of NAFLD, mainly by improving peripheral insulin resistance [139].

Taken together, the restriction of alcohol intake could be an effective measure for the prevention of NAFLD. In addition, it is encouraged that all people should minimize alcohol intake and adhere to American Dietary Guidelines, which recommend restricting alcohol use to one drink per day for women, two drinks per day for men and that people with severe liver disease should abstain from alcohol [140]. More prospective studies are needed in the future to focus on the mechanisms by which alcohol plays a role in NAFLD prevention, as well as to determine specific alcohol intakes.

#### Soft drinks

Soft drinks (SD) are carbonated beverages, which are sweetened with monosaccharides, in particular fructose and glucose, or their progenitor sucrose [141]. Furthermore, cola SD contain caramel coloring with high levels of advanced glycation end products, which could increase insulin resistance and inflammation [142]. Moreover, it has been shown that the consumption of SD could increase the prevalence of NAFLD independently of metabolic syndrome [143]. Additionally, it was demonstrated that individuals who drank > 1 cup/day had a higher prevalence of metabolic syndrome than those who drank < 1 cup/day (OR: 1.48, 95% CI: 1.30–1.69) [144]. Therefore, restricting the intake of SD may be a key strategy in preventing NAFLD, as the potential mechanisms of action involve insulin resistance and oxidative stress.

### Prophylactic effects of dietary strategies on NAFLD

#### Mediterranean diet

Mediterranean diet (MD) is currently the only recommended potential treatment for patients with NAFLD [145]. The composition of the diet includes 35% to 45% of the energy intake from fat, 35% to 40% carbohydrate and 15% to 20% protein [146]. Moreover, MD is characterized by olive oil (20–30 g/d) as the basic source of fat, mainly eating vegetables, whole grains, fruits and nuts [147]. Additionally, MD has a high ratio of mono-unsaturated fatty acids to SFA (approximately 2 : 1) with a total fat accounting for 30% to 40% of daily energy consumption [146].

Indeed, several studies have consistently shown that the MD significantly reduces NAFLD-related risks by improving lipid profiles, reducing hepatic fat content, enhancing insulin sensitivity, and decreasing intrahepatic fat and the characteristics and results of these relevant studies are shown in Table 1 [148–152]. Moreover, MD has been shown to increase insulin sensitivity, decrease hepatic steatosis, and reduce NASH [153]. Besides, MD is recommended for NAFLD patients because most of its components are negatively correlated with the prevalence, severity, or regression of NAFLD [148].

**Table 1.** Characteristics and results of relevant studies validating the effect of the mediterranean diet on adults with NAFLD.

Author, year	Study type	Country	Study duration	No. of participants	Study aim	Component foods	Intervention	Main results
Ludovico et al. 2017 [148]	Prospective	Italy	6 months	50	Compare the effects of the MED diet on overweight patients with NAFLD	Group A: high consumption of olive oil, vegetables, fruits, legumes, whole grains, moderate fish and dairy, low red/processed meat. Group B included additional antioxidant-rich foods and supplements	Group A: moderately hypocaloric MED diet Group B: moderately hypocaloric MED diet and antioxidant supplement daily Group C: no intervention	Group A and group B improve anthropometric parameters, lipid profiles and reduce liver fat accumulation and liver stiffness Group B showed a significant improvement in insulin sensitivity
Yftach et al. 2019 [149]	Randomized controlled trial	Israel	18 months	278	Investigate if reduction in HFC is a major mediator of the cardiometabolic benefit of lifestyle intervention	Low-fat diet: whole grains, vegetables, fruits, and legumes The MED/LC diet: vegetables and legumes and low in red meat, with poultry and fish replacing beef and lamb.	Participants were randomized to low-fat or Mediterranean/low-carbohydrate (MED/LC + 28 g walnuts/day) diets with/without moderate physical activity	MED/LC diet could be more effective in reducing HFC percentages MED/LC diet induce a greater decrease in hepatic fat content than low-fat diet
Yaskolka et al. 2021 [150]	Randomized controlled trial	Israel	18 months	294	Examine the effectiveness of green MED diet, further restricted in red/ processed meat, and enriched with green plants and polyphenols on NAFLD, reflected by IHF loss	Both isocaloric MED groups consumed 28 g/day walnuts. The green-MED group further consumed green tea (3–4 cups/day) and Mankai (a Wolffia globosa aquatic plant strain; 100 g/day frozen cubes) green shake	Group A: HDG + PA + standard nutritional counselling Group B: MED + PA + 28 g/day walnuts (+ 440 mg/day polyphenols provided) Group C: Green-MED group + PA + 28 g/day walnuts + green tea (3–4 cups/day) and Mankai (a Wolffia globosa aquatic plant strain; 100 g/day frozen cubes) green shake (+ 1240 mg/day total polyphenols provided)	The prevalence of NAFLD declined to: 54.8% (HDG), 47.9% (MED) and 31.5% (Green-MED), *P* = 0.012 between groups The Green-MED diet, amplified with green plant-based proteins/polyphenols as Mankai, green tea, and walnuts, and restricted in red/processed meat can double IHF loss than other healthy nutritional strategies and reduce NAFLD in half
Ruth et al. 2015 [151]	Retrospective	Hong Kong	NA	797	Examine the association of two diet-quality scores (MDS and DQI-I) with NAFLD prevalence in 797 Chinese aged 18 years or above	Vegetables and legumes, fruits and dried fruits, as well as vitamin C	Volunteers were given two patterns of international dietary quality index DQI-I and MDS to observe the effects of these two diets on the incidence of NAFLD	A better diet quality characterized by higher DQI-I score and higher consumption of vegetables, legumes, and fruits is associated with a lower likelihood of NAFLD among Chinese adults in Hong Kong
Kawaguchi et al. 2021 [152]	Meta-analyses	Japan	6 weeks to 6 months	250	Investigate the effects of the MED diet on hepatic steatosis and insulin resistance in participants with NAFLD	A high intake of plant-based foods and olive oil and low intake of dairy products	MED vs no intervention or other diets such as low-fat diets	MED diet significantly reduced FLI compared with the control diet (SMD: −1.06, 95% CI: −1.95 to −0.17, *P* = 0.02); Med diet significantly reduced HOMA-IR compared with the control diet (SMD: −0.34, 95% CI: −0.65 to −0.03, *P* = 0.03)

HFC, hepatic fat content; MED/LC, Mediterranean/low-carbohydrate; NAFLD, non-alcoholic fatty liver disease; IHF, intrahepatic fat; HDG, healthy dietary guidelines; NA, not available; MDS, Mediterranean Diet Score; DQI-I, Diet Quality Index-International; FLI, fatty liver index; SMD, standard mean difference; HOMA-IR, homeostasis model assessment of insulin resistance.

To sum up, the prophylactic impact of MD on NAFLD is well established. However, due to the slightly different cultures, religions and agriculture of the countries, the structure of MD has significant differences from region to region, and consequently the effectiveness of various MD in preventing NAFLD may vary [154]. Therefore, further investigations are required to study the differences in MD patterns in different regions and countries, and enable to explore a dietary structure of MD that is most suitable for the prevention of NAFLD.

#### Intermittent fasting

Intermittent fasting (IF) could be defined in the simplest terms as alternating eating and feeding [155]. Besides, there are many common patterns of IF, the most common of which are three types: alternate-day fasting, where individuals fast every other day; 5 : 2 fasting, which involves eating normally for five days and restricting calorie intake to about 500–600 calories on the other two non-consecutive days; and time-limited fasting, which limits eating to a specific window each day, such as 8 h, followed by 16 h of fasting [156]. Typically, the maximum period of food restriction in IF practices range up to 3 weeks, depending on the specific regimen and individual tolerances [157]. In the past few years, IF diets have become very popular because IF does not require calculating calorie intake compared to traditional dieting modalities such as calorie restriction [158]. However, it is important to note that IF does not permit excessive caloric intake during feeding periods, as this could negate the potential benefits of the fasting regimen.

Moreover, it was suggested that IF could facilitate weight loss and improve cardiac metabolism, particularly in overweight or obese adults, and may therefore be a viable preventive measure for NAFLD [159,160]. Furthermore, a meta-analysis has discovered that implementing a modified alternate-day fasting regime for a period of 1–2 months could result in a substantial decrease in body mass index for both healthy individuals and those who are overweight, obese, or suffer from NAFLD [161]. Besides, it was reported that alternate-day fasting and 5 : 2 diet result in similar degrees of weight loss (4% to 8% reduction from baseline) in obese patients in the short term (8–12 weeks), which is benefit to the prevention of the NAFLD [162–164]. In addition, IF improves and protects the body state by triggering a ‘metabolic switch’ from liver-derived glucose to adipocyte-derived ketone bodies, triggering evolutionarily conserved and adaptive cellular responses in animals, such as reducing anabolism, enhancing stress resistance, promoting mitochondrial biogenesis, improving glucose and lipid metabolism, and enhancing antioxidant defense [160,165].

However, IF is not suitable for everyone, and it is not recommended for certain people, including children under the age of 12, people over the age of 70 and pregnant women [156]. The prevalence of NAFLD in children has been reported to be approximately 7.6% in the general pediatric population and up to 34.2% in those with obesity [166]. This group is particularly vulnerable due to ongoing growth and development, which can be negatively impacted by fasting regimens. Among individuals aged 70 and above, the prevalence of NAFLD exceeds 25%, often compounded by age-associated sarcopenia and malnutrition risks, making IF inappropriate [167]. Pregnant women require consistent nutrient intake to support fetal development, rendering IF unsuitable for this population. To date, there hasn’t been a study comparing the three IF regimens mentioned above, so it remains uncertain which IF regimen is most advantageous for preventing NAFLD.

In conclusion, IF may have potential as a useful intervention for NAFLD and further studies are needed to precisely evaluate the mechanism, human efficacy, target population, and safety of IF.

#### The ketogenic diet

The ketogenic diet (KD) is characterized by a macronutrient distribution of approximately 70–80% fat, 10–20% protein and 5–10% carbohydrate and carbohydrate intake of less than 20–50 g/day [168]. Moreover, KD could be divided into two categories, very low-calorie diet (carbohydrate intake to < 50 g/day and caloric intake < 800 kcal/day) and very low carbohydrate diet (carbohydrate intake to < 50 g/day and energy intake typically > 1000 kcal/day) [169,170].

KD reduces insulin levels due to its very low carbohydrate content, resulting in an increase in fatty acid oxidation and a decrease in lipogenesis, which is an important goal of NAFLD prevention [171]. Besides, when people on a KD, the body metabolizes more ketones (including acetoacetic acid, β-hydroxybutyric acid, and acetone) to use as an energy source [172]. Furthermore, it was found that KD could significantly reduce hepatic fat content and hepatic insulin resistance, these changes were associated with increased net hepatic triglyceride hydrolysis as well as decreased endogenous gluconeogenesis and serum insulin concentrations in a mouse model [173]. Likewise, there is evidence that hitherto undescribed adaptations underlie the reversal of NAFLD by KD and highlight hepatic mitochondrial flux and redox status as potential therapeutic targets for NAFLD [174]. In addition, it was demonstrated that the isocaloric KD of increased protein content in obese patients with NAFLD and found a reduction in liver fat (mean reduction 43.8%) in a prospective study (*n* = 17) [175].

Additionally, KD prevented NAFLD in a time-dependent manner and 2 weeks of KD was the optimal time point for alleviating NAFLD [176]. However, KD also has various side effects, such as vomiting, headache, fatigue, constipation, irritability, and drowsiness, which cannot be ignored [177]. In addition, the energy intake of the KD is very strict and this method is difficult to maintain in the long term [177].

Collectively, KD is a preventive tool that could be used to address steatosis degeneration, weight loss, and high cholesterol levels. However, whether all NAFLD patients could safely use KD over the long term remains largely unknown. Therefore, large-scale randomized controlled trials are needed to determine the impact of KD on NAFLD and long-term safety.

#### Dietary approaches to stop Hypertension diet

The Dietary Approaches to Stop Hypertension (DASH) diet, also known as the termination of hypertensive die, differs from the MD in its specific focus on reducing sodium intake and emphasizing low-fat dairy products, while the MD prioritizes olive oil and a high intake of monounsaturated fats [178]. This diet is characterized by a high intake of fruits, vegetables, whole grains, and low-fat products and it is low added sugar, sugary drinks and red and processed meat [179].

Due to DASH improves glucose and lipid metabolism, contributes to weight loss, and reduces cardiovascular risk, it has attracted the interest of numerous researchers as a potential dietary strategy for preventing NAFLD [180,181]. It was confirmed that adherence to the DASH diet was inversely associated with the risk of NAFLD (OR: 0.0.70; 95% CI: 0.61–0.80); however, and the association was not significant after adjustment for body mass index (BMI) and dyslipidemia (OR: 0.92; 95% CI: 0.73–1.12) [182]. Moreover, a cross-sectional analysis (*n* = 3051) found that adherence to the DASH diet was independently associated with a significant reduction in the prevalence of NAFLD and reductions in levels of inflammation, insulin resistance, and BMI (*P* = 0.009) [183]. In addition, a meta-analysis evaluating randomized trials concluded that DASH diet may enhance insulin sensitivity regardless of weight loss, mainly when prescribed for more than 16 weeks [184].

In summary, the current findings suggested that DASH diet helps to reduce BMI, correct metabolic disorders, and improve the degree of hepatic steatosis, thereby reducing the risk of NAFLD. Therefore, it is believed that further studies are needed to determine the potential benefit of DASH diet in NAFLD patients.

### Prophylactic effects of exercise intervention on NAFLD

Regular exercise is defined as sustained moderate-intensity physical activity including walking, jogging, bicycling, and swimming, usually for 60 min per day, 3–5 times per week [185]. Moreover, regular exercise could be considered an effective strategy in the current prevention of NAFLD, as exercise training is not only an inexpensive intervention with therapeutic and preventive value, but also reduces the risk factors for cardiovascular disease in NAFLD, such as diabetes and hypertension [186]. A systematic review reported that increase physical activity is negatively associated with NAFLD incidence [187]. Besides, different forms of exercise such as aerobic, resistance, or high intensity intermittent exercise seem to have similar effects on liver fat [188]. Furthermore, an experiment showed that moderate-intensity exercise training 30–60 min, 5 times a week for 16 weeks, could reduce liver fat content by about 10%, but in the absence of weight loss, and did not increase triglyceride secretion rate [189]. Additionally, three months of aerobic exercise, including brisk walking/jogging or rhythmic aerobic exercise, respectively resulted in a 47% and 48% reduction in alanine transaminase and aspartate aminotransferase [190]. Although the US Department of Health and Human Services exercise recommendations (moderate intensity for 30–60 min 5 times a week for a 16-week period) may reduce approximately 10% decrease in liver fat, it was suggested that more rigorous exercise protocols combined with dietary intervention may be required to induce improvements in liver histological features associated with NASH [191].

Exercise could alleviate NAFLD through multiple mechanisms of action. Exercise increases peripheral insulin sensitivity, but has little effect on liver insulin sensitivity, resulting in a net improvement in insulin metabolism [192]. Moreover, it was found that exercise reduced lipid droplets formation, decreased hepatic triglyceride in the liver induced by high-fat diet *in vivo* and *in vitro* models [193]. Of note, exercise improves NAFLD by reducing intrahepatic fat content, increasing β-oxidation of fatty acids, inducing hepato-protective autophagy, overexpressing PPAR-γ, as well as attenuating hepatocyte apoptosis and increasing insulin sensitivity [194]. Besides, exercise training also suppresses reactive oxygen species overproduction and oxidative stress in NAFLD *via* up-regulation of several antioxidant enzymes and anti-inflammatory mediators [195].

In conclusion, physical activity may play an important role in the prevention of NAFLD and regular exercise is beneficial in reducing the risk of NAFLD and more studies are needed in the future to explore the mechanism of combined diet and exercise intervention and provide feasible recommendations for relevant individuals.

### Prophylactic effects of lifestyle on NAFLD

Modern lifestyle may have a potential impact on the development of NAFLD. Sedentary behavior, smoking and second-hand smoke exposure, sleep quality, and frequency of tooth brushing are all common lifestyle factors that can influence the development of NAFLD.

#### Sedentary behavior

Sedentary behavior is rapidly emerging as a global issue that has detrimental effects on public health [196,197]. Sedentary behaviors include sitting or lying, and not consuming enough energy during waking hours [198]. Besides, several epidemiological studies have shown that sedentary behavior is strongly associated with risk factors for NAFLD such as obesity, diabetes, insulin resistance, and metabolic disorders [199–201].

Previously, a cross-sectional study (*n* = 2054) found a positive association between sitting time (> 7.1 h/day) and the prevalence of NAFLD (OR: 1.09; 95% CI: 1.04–1.67) [202]. Furthermore, there is evidence that prolonged sitting time and reduced physical activity levels were positively associated with the prevalence of NAFLD (*P* < 0.001), supporting the importance of reducing sitting time in addition to promoting physical activity [203]. In addition, it has been observed that sedentary time has adverse effects on NAFLD (*P* = 0.0002), which mainly mediated by increased fat mass or decreased skeletal muscle mass [204].

In conclusion, engaging in physical activity and reducing sedentary time may present a novel solution for preventing NAFLD. Even small, non-exercise movements such as standing, walking short distances, or stretching during the day could contribute to mitigating risks associated with prolonged inactivity and reducing the overall burden of sedentary behavior.

#### Smoking and secondhand smoke

There are some studies have shown that smoking is also associated with increased prevalence and incidence of liver disease [205,206]. A cross-sectional study (*n* = 8580) found that passive smoking and heavy active smoking were associated with prevalent NAFLD among middle-aged and older Chinese (OR = 1.25, 95% CI: 1.05–1.50) [207]. Furthermore, a 31-year cohort study involving 1315 participants shown that passive smoking in the lives of children and adults associated with increased risk of fatty liver (relative risk = 1.99, 95% CI: 1.14–3.45) [208]. In addition, compared with non-smokers, the OR of NAFLD in former smokers and current smokers was 1.12 (95% CI: 0.90–1.41) and 1.38 (95% CI: 1.08–1.76), respectively [209]. Notably, ex-smokers who ceased smoking for < 10 years (OR = 1.33, 95% CI: 1.00–1.77) were more likely to have a strong correlation with NAFLD [209]. Moreover, there is also evidence that long-term smoking cessation led to a significant reduction in insulin resistance in asymptomatic Korean male smokers [210].

Overall, these researches provide evidences of a possible association between smoking and NAFLD development. Therefore, doctors should warn patients to reduce smoking to prevent NAFLD based on the potential impact of smoking on the pathogenesis and progression of NAFLD. Furthermore, patients with NAFLD should be encouraged to quit smoking when they are informed of the potential of secondhand smoke causing NAFLD among their family members.

#### Sleep

In modern life, staying up late has become a new normal for many young people [211]. Several studies have reported that staying up late frequently increases the risk of fatty liver [212–214]. Moreover, it was found that staying up late was associated with a higher risk of NAFLD (OR = 1.37, 95% CI: 1.10–1.70), and the risk of fatty liver gradually decreased with increasing healthy sleep scores [214]. Furthermore, according to a bidirectional two-sample Mendelian randomization study (*n* = 1029), it was demonstrated that difficulty getting up in the morning and usually insomnia were associated with an increased risk of NAFLD (hazard ratio [HR]: 1.51, 95% CI: 1.27–1.78 and HR: 1.46, 95% CI: 1.21–1.75, respectively) [215]. In addition, a review has summarized the epidemiology and pathophysiological mechanisms of sleep on NAFLD and found that interrupted sleep could further promote the progression of liver disease [216].

There are various factors contribute to poor sleep patterns, such as working night shifts which disrupt the circadian rhythm, and excessive screen time, which suppresses melatonin production [217]. These habits not only reduce overall well-being but also heighten the risk of NAFLD by disturbing metabolic and hormonal balances [218]. In contrast, practicing good sleep hygiene could significantly improve sleep quality and reduce NAFLD risk [219]. This includes maintaining a consistent sleep schedule, creating a comfortable sleep environment, and limiting screen time before bedtime. Moreover, the ideal sleep duration for adults is 7–9 h per night, accompanied by consistent sleep and wake times to ensure physical and mental recovery [220].

In conclusion, unhealthy sleep habits are associated with an increased risk of NAFLD and high sleep quality is associated with a decreased risk of NAFLD. Therefore, it is necessary to improve the public’s health awareness of sleep disorders to comprehensively manage the risk of NAFLD.

#### Tooth brushing

The present studies suggested that higher frequency of brushing is negatively correlated with the risk of NAFLD [221,222]. A retrospective longitudinal study (*n* = 25804) discovered that individuals who brushed their teeth 1–2 times a day or 3 times a day had a lower risk of NAFLD (OR: 0.85, 95% CI: 0.77–0.95 and OR: 0.74, 95% CI: 0.67–0.82, respectively) than those who brushed their teeth once or less than once a day [221]. Besides, it was shown that the risk of NAFLD was lower in the group who performed toothbrushing more than 3 times a day compared to the group that performed toothbrushing less than once a day (OR: 0.56, 95% CI: 0.35–0.90) in a retrospective study (*n* = 6352) [222].

However, the effect of brushing teeth may vary depending on the type of oral hygiene product used. Among the herbal agents, miswak extract and neem extract were the most commonly used and they often incorporate natural antimicrobial and anti-inflammatory properties that could enhance oral microbiota balance and reduce systemic inflammation [223]. Conversely, manufactured toothpastes commonly contain fluoride and synthetic antimicrobials, which may have different mechanisms of action [224].

In summary, there is a negative relationship between the frequency of tooth brushing and the development of NAFLD. Therefore, tooth brushing should be considered as a potential preventive therapy for NAFLD.

### Prophylactic effects of social environment on NAFLD

Social environment includes socio-economic and cultural factors and it also related to the prevalence of NAFLD, but their exact role is debated [225,226]. Moreover, it was found that higher education (those with a college or university degree) was associated with reduced risk of NAFLD [225]. However, there is evidence that acculturation, education level, health-care use and income were not found to be independently associated with risk of developing NAFLD, which suggesting that environmental factors might play a role on a background of genetic predisposition [226]. Therefore, further clinical trials are needed to prove whether social environmental factors could have a positive effect on the prevention of NAFLD.

### Prophylactic effects of natural environment on NAFLD

#### PM_2.5_

Previous studies suggested that exposure to ambient air particulate matter (PM_2.5_) is associated with an increased risk of NAFLD [227,228]. High levels of PM_2.5_ are often due to industrial emissions, vehicular exhaust, biomass burning, and other urban activities [229]. PM_2.5_ exposure levels above the WHO air quality guideline of 5 µg/m³ (annual mean) or the interim target levels of 15 µg/m³ for high-pollution regions are considered unhealthy.

Additionally, it was concluded that PM_2.5_ exposure could promote the development of NAFLD by disrupting intestinal epithelial and microbial homeostasis, triggering endoplasmic reticulum stress, and inducing abnormal expression of specific microRNAs or inflammatory factors [230]. Furthermore, PM_2.5_ exposure was demonstrated to exacerbate insulin resistance, impair glucose tolerance, induce peripheral inflammation, and promote atherosclerosis in a mouse model [231]. Besides, it also demonstrated that PM_2.5_ inhalation leads to hepatic oxidative stress and inflammatory reaction, resulting in abnormal liver function, thereby promoting hepatic lipid accumulation [231]. There is increasing evidence that PM_2.5_ is positively associated with the risk of NAFLD and progressive fibrosis, with a one standard deviation (7.5 µg/m^3^) increase in PM_2.5_ exposure, increasing the risk of NAFLD by 10% (95%CI: 0.09–0.11) and the risk of progressive fibrosis by 8% (95% CI: 0.07–0.09) [232].

In conclusion, it is suggested that implementing policies to limit air pollutant emissions could help prevent NAFLD. Moreover, the high risk of NAFLD groups should pay attention to the air quality of their living areas and rationally plan outdoor activities to reduce exposure to air pollution [228]. Furthermore, researches on the relationship and its potential mechanism between NAFLD and PM_2.5_ exposure progression are very limited and further large-scale epidemiological investigations are needed.

#### Polyfluoroalkyl substances

Polyfluoroalkyl substances (PFAS) and polychlorinated biphenyls, which are present in a variety of industrial and consumer products, have been shown to be associated with the incidence of NAFLD [233]. Due to PFAS are highly persistent in the environment, the general population is widely exposed to them, primarily through diet and contaminated water [234]. Besides, PFAS and polychlorinated biphenyls has been shown to promote hepatic steatosis/triglyceride accumulation and liver injury through the generation of reactive oxygen species [235]. Moreover, it was reported that PFAS are highly hepatotoxic, interfering with glucose and lipid metabolism and increasing liver enzymes, all of which are major factors influencing the increased incidence of NAFLD [236]. In addition, researchers have demonstrated that exposure to PFAS appears to be associated with perturbations in hepatic metabolic pathways, particularly bile acid and lipid metabolism, and that women may be more sensitive to the harmful effects of PFAS [234].

In summary, PFAS and polychlorinated biphenyls exposure may increase risk of NAFLD. Therefore, enhancing our understanding of how environmental factors impact NAFLD and its mechanism is essential for devising new, effective prevention strategies against NAFLD.

### The interaction between sarcopenia and NAFLD

Sarcopenia, the loss of skeletal muscle mass and function, has emerged as a significant factor influencing the severity and progression of NAFLD [237]. There was evidence that low muscle mass is associated with histological severity in NAFLD, and sarcopenia is significantly associated with NASH and significant fibrosis [238]. Besides, it was reported that not only obesity-related NAFLD is closely related to sarcopenia, but also the pathological features of lean NAFLD such as visceral obesity, insulin resistance, and metabolic inflammation are also predisposing factors for sarcopenia, and the loss of muscle mass and function further aggravates ectopic fat accumulation and lean NAFLD [239].

There is no standardized treatment for sarcopenia due to NAFLD, other than regular physical activity and a balanced diet, but promising remedies and novel substances appear on the scene [240]. For instance, nutritional strategies, particularly those focused on protein supplementation and vitamin D, are critical for preserving muscle tissue and mitigating liver inflammation [241].

In summary, NAFLD could be associated with sarcopenia, and experiments have shown that there are multiple pathogenic mechanisms that could explain this association. Therefore, more researches are needed in the future to explore a drug to co-target NAFLD and skeletal muscle reduction.

## Summary of established and unresolved factors

**Figure 1.**
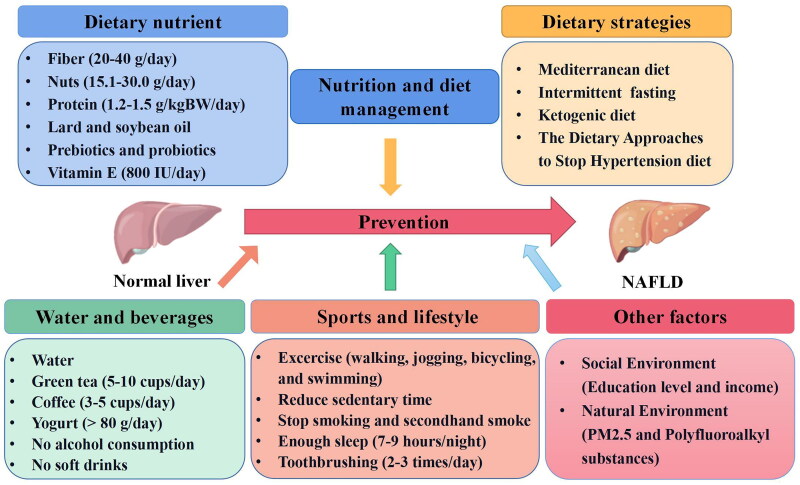
The role of prophylactic nutrition, dietary strategies, exercise, lifestyle and the environment in the management of NAFLD. (images: Flaticon.com, by figdraw).

This review summarized the role and mechanism of nutrition, dietary strategies, exercise, lifestyle and environment in the prevention of NAFLD and made feasible recommendations (The relevant information is shown in Figure 1). Firstly, individuals could fortify their defenses against NAFLD onset and progression by meticulously managing nutrient intake, encompassing both macronutrients and micronutrients. Moreover, the adoption of specific dietary strategies including the Mediterranean diet, intermittent fasting, ketogenic diet, and the Dietary Approaches to Stop Hypertension diet offer promising avenues for mitigating the pathological trajectory of NAFLD. Besides, as the complementary to dietary interventions, engaging in walking, jogging, bicycling, and swimming holds immense potential for attenuating NAFLD risk factors, particularly by modulating body weight and metabolic parameters. In addition, embracing a holistic and health-oriented lifestyle including reduce sedentary, no smoking, good sleep not only mitigates the risk of NAFLD but also fosters comprehensive prevention and control measures against metabolic disorders. Finally, the potential role of social and environmental factors in the prevention of NAFLD is also highlighted. Additionally, the progress of NAFLD is related to a variety of risk factors (The relevant studies are shown in Table 2). Therefore, more studies are needed in the future to study the effects of different risk factors on NAFLD.

**Table 2.** The risk factors for NAFLD.

Conditions with established association	Conditions with emerging association
Diabetes mellitus (type2)	Genetic (PNPLA3 and TM6SF2 variants)
Obesity	Low birth weight
High blood pressure	BMI and plasma insulin levels
Metabolic syndrome	Polycystic ovary syndrome
Virus infection	Hypopituitarism
Alcohol consumption	Sarcopenia

## Conclusion

Through a comprehensive and careful review of the existing literature, this review synthesizes current evidence on nutrition, dietary patterns, exercise, and lifestyles that affect NAFLD, and provides robust and detailed prevention recommendations for people at high risk of NAFLD. Furthermore, the review also focuses on the social and natural environment which was less discussed previous. These findings provide valuable insights for future research work and guide clinical practice, thus promoting our joint efforts in addressing the increasing burden of NAFLD on global public health.

However, this review also has some limitations in the review process. Firstly, there is still a lack of high-quality, large-sample, multi-center studies in this topic and thus further validation by subsequent relevant experiments is needed. Additionally, it is essential to explore the interaction between genetic predisposition and lifestyle factors to deepen our understanding of the underlying mechanisms in the future studies. Moreover, such research should also aim to develop personalized prevention strategies tailored to high-risk groups. These efforts are crucial to provide robust evidence for preventive measures and to mitigate the global burden of NAFLD.

## Data Availability

Data sharing is not applicable to this article as no new data were created in this study.
